# A patient with relapsed high-grade serous ovarian carcinoma with somatic *RAD51C* mutations treated with PARPi monotherapy: a case report

**DOI:** 10.20517/cdr.2022.12

**Published:** 2022-06-22

**Authors:** Siew-Fei Ngu, Hextan Y. S. Ngan, Karen K. L. Chan

**Affiliations:** Department of Obstetrics and Gynaecology, The University of Hong Kong, Hong Kong, China.

**Keywords:** Ovarian cancer, PARP inhibitor, *BRCA*, *RAD51C*

## Abstract

We report our experience in the management of a relapsed ovarian cancer patient with somatic *RAD51C* mutation, treated with olaparib monotherapy. The patient was diagnosed with stage 4 high-grade serous ovarian carcinoma and was treated with neoadjuvant chemotherapy, cytoreductive surgery, and postoperative chemotherapy. After a second cancer recurrence, she underwent FoundationOne CDx testing following disease progression on multiple lines of chemotherapy. Based on the FoundationOne CDx results, olaparib monotherapy was started. After 13 months of therapy, all lesions responded to the treatment, and she achieved complete response as demonstrated by normalization of the levels of CA125 and positron emission tomography-computed tomography (PET-CT). We plan to continue olaparib monotherapy until disease progression.

## INTRODUCTION

Recommended treatment for patients with relapsed ovarian cancer depends on platinum-free intervals. Generally, patients who have recurrence after 6 months of completion of platinum-based chemotherapy are considered platinum-sensitive, while those who recur within six months are considered platinum-resistant. Patients with platinum-sensitive relapse are typically treated with platinum-based regimens, while those with platinum-resistant disease are often given second-line single-agent chemotherapy such as gemcitabine, pegylated liposomal doxorubicin, weekly paclitaxel, or topotecan. It is well recognized that the time to progression usually shortens with subsequent relapses, so that patients encounter shorter treatment-free intervals. Following consecutive therapies, the median duration of progression-free survival often leaves patients with limited or no treatment-free interval and accumulating toxicities.

*BRCA1* and *BRCA2* genes are the most identified germline mutations in patients with ovarian cancer, and their proteins are important for DNA repair. Mutations in these genes lead to homologous recombination (HR) deficiency, which may be targeted in the treatment of ovarian cancer with poly ADP-ribose polymerase inhibitors (PARPi)^[1]^. Among other HR-related genes which have been found to significantly increased the ovarian cancer risk are *RAD51C *and* RAD51D*, coding for proteins that support the process of DNA repair^[2]^. In epithelial ovarian cancers, *RAD51C *and* RAD51D* mutations were found in 0.41%-0.68% and 0.35%-1.13% of patients, respectively^[2-4]^. Tumors with mutations in *RAD51C* and *RAD51D* have been found to be *BRCA*-like with high genomic loss of heterozygosity and responded to PARPi (rucaparib) at similar rates to *BRCA*-mutated disease^[5]^.

PARPi are a type of targeted cancer treatment that block the protein PARP, an enzyme that assists in the repair of damaged DNA. In cancer treatment, blocking PARP may help prevent cancer cells from repairing their damaged DNA, leading to cell death^[5]^. Several PARPi have been approved by the US Food and Drug Administration (FDA) for the treatment of ovarian cancer including olaparib, niraparib, and rucaparib.

We report our experience in the management of a relapsed ovarian cancer patient with somatic *RAD51C* mutation, treated with olaparib monotherapy.

## CASE REPORT

### History, presentation, and initial treatment

A 74-year-old woman first presented in October 2016 with abdominal pain and distension. She had a history of breast cancer treated with lumpectomy and adjuvant radiotherapy in 2009 and autoimmune hypothyroidism. Pelvic ultrasound showed a 7 cm mass in the Pouch of Douglas and gross ascites. Tumor marker evaluations showed elevated levels of cancer antigen (CA) 125 (1762 U/mL). Paracentesis was performed and peritoneal fluid cytology showed metastatic adenocarcinoma with immunohistochemical markers favoring female genital tract origin (CK7 and PAX8 positive, CK20 negative). Subsequent positron emission tomography-computed tomography (PET-CT) scan showed extensive hypermetabolic lesions in the peritoneum and bilateral adnexa with metastasis to lymph nodes, liver, and spleen, consistent with stage 4 ovarian cancer. After four cycles of neoadjuvant carboplatin and paclitaxel, CA125 decreased to 60 U/mL. PET-CT showed good partial response: previous peritoneal, nodal, liver, and spleen metastases had all resolved, and there was a reduction in the size of the adnexal mass. She then underwent interval debulking surgery including total abdominal hysterectomy, bilateral salpingo-oophorectomy, and omentectomy in January 2017. There was some miliary disease left on the mesentery after the debulking surgery. Histology showed high-grade serous carcinoma involving both ovaries, omentum, and peritoneum. The patient received 6 cycles of post-surgical chemotherapy comprising cisplatin and paclitaxel until July 2017. Carboplatin was substituted with cisplatin due to thrombocytopenia. PET-CT performed upon completion of chemotherapy showed complete response and CA125 also normalized (11.1 U/mL). Germline *BRCA1/2* mutation test was negative.

### Chemotherapy for recurrence

The patient had the first disease relapse seven months later, in February 2018. PET-CT showed recurrence in the peritoneum, liver, and lymph nodes. She was treated with 6 cycles of cisplatin, gemcitabine, and bevacizumab, due to which she had recurrent grade 4 thrombocytopenia and grade 3 anemia requiring platelet and blood transfusion during the course of the chemotherapy. Following 6 cycles of chemotherapy, PET-CT in September 2018 showed a complete response. She then received 11 more cycles of maintenance bevacizumab until May 2019. In June 2019 (nine months after completion of the last chemotherapy), she had a second relapse with nodal lesions in the abdomen and left supraclavicular fossa. In view of poor marrow reserve, advanced age, and previous chemotherapy, she was prescribed pegylated liposomal doxorubicin instead of platinum-based chemotherapy. Subsequently, the disease progressed despite multiple lines of chemotherapy including four cycles of pegylated liposomal doxorubicin, 6 cycles of topotecan, and five cycles of docetaxel until September 2020. PET-CT in October 2020 showed hypermetabolic lymphadenopathies in the abdomen, thorax, and left supraclavicular regions and liver metastasis.

### PARPi monotherapy

**Figure 1:**
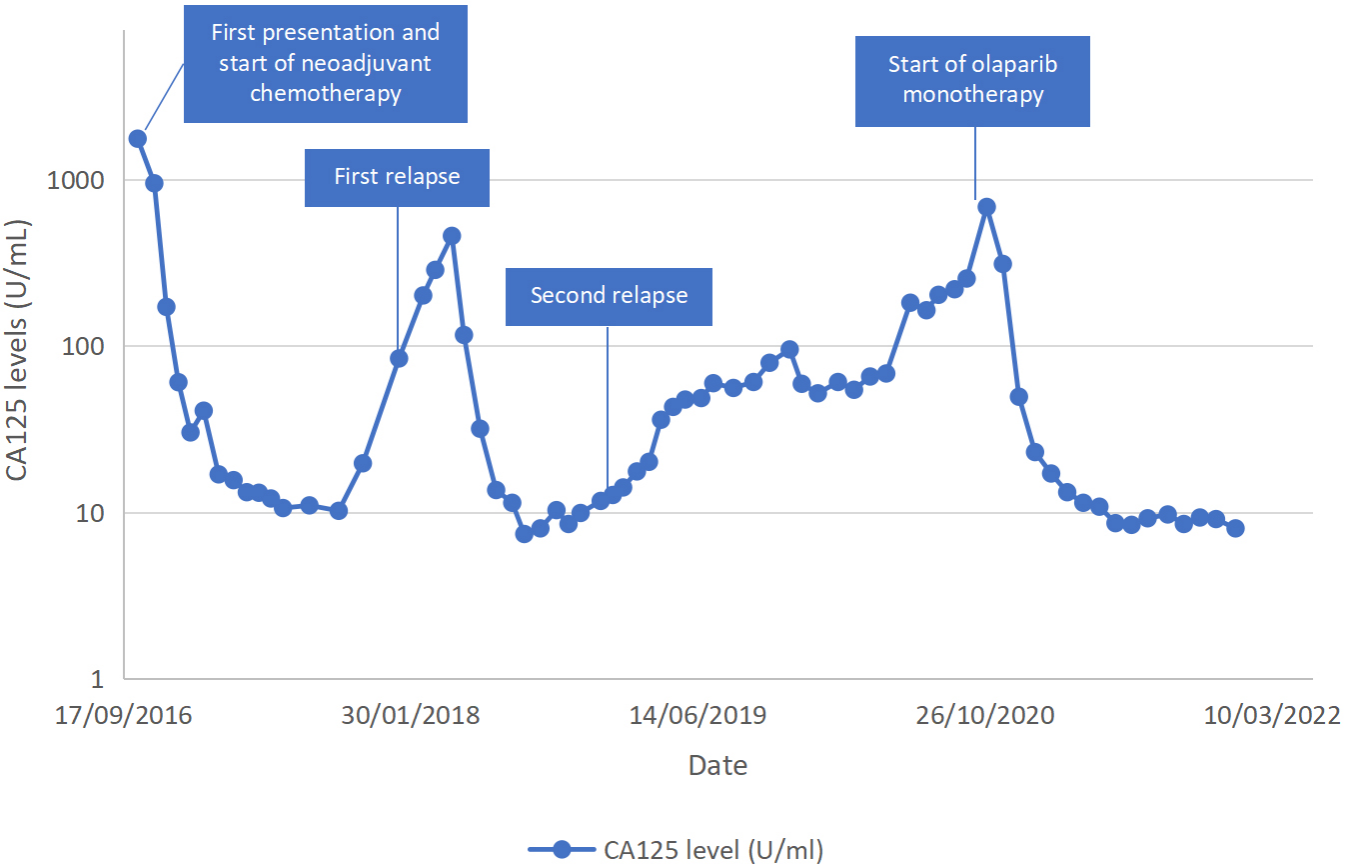
The blood levels of tumor marker CA125 of the patient.

She then underwent FoundationOne CDx^TM^ (Foundation Medicine, Inc) testing, which found *RAD51C* mutations in the tumor. Testing for germline *RAD51C* had not been done due to limited availability for testing in our setting. The patient was started on 300 mg olaparib monotherapy twice daily in October 2020. The CA125 just before the commencement of olaparib was 685 U/mL. Due to grade 3 anemia (Hb 7.9 g/dL), the dose of olaparib was reduced to 200 mg twice a day in February 2021. The levels of CA125 gradually decreased and normalized, with the latest results of 9 U/mL [Figure 1]. PET-CT in November 2021 showed a complete response. Last seen in December 2021, the patient was well and continued olaparib therapy.

## DISCUSSION

Recently, PARPi have significantly changed the landscape of treatment for advanced and relapsed ovarian cancer. Olaparib, a PARPi, has demonstrated antitumor activity among patients with relapsed ovarian cancer who have a *BRCA1/2* mutation^[6,7]^. Olaparib monotherapy is approved by the US Food and FDA for the treatment of patients with relapsed ovarian cancer who have a germline *BRCA* mutation and have received three or more prior lines of chemotherapy. In pooled data from 6 trials, the overall response rate of olaparib was 31% in this group of patients, with responses seen in both platinum-sensitive and platinum-resistant diseases^[8]^. In a phase II trial (ARIEL2, a single-arm, open-label study of the PARPi rucaparib in relapsed high-grade ovarian carcinoma), *RAD51C *and *RAD51D* mutations were associated with the clinically meaningful activity of rucaparib similar to that of *BRCA* mutations. Of 206 patients, there were four patients with *RAD51C *mutations, of whom three patients had a partial response and one patient had stable disease with rucaparib therapy^[5,9]^. Furthermore, a case report on a heavily pretreated patient with ovarian carcinosarcoma harboring a germline *RAD51D* mutation demonstrated excellent and durable partial response with olaparib monotherapy^[10]^. Extrapolated from this information and the suggestion from the FoundationOne CDx report of our patient, she was given olaparib monotherapy after she failed to respond to multiple lines of chemotherapy. Olaparib was used on our patient as rucaparib was not available locally. At the time of writing, our patient has been on olaparib monotherapy for 14 months and tolerated it well. To our surprise, all lesions responded to the treatment, and she achieved complete response after 13 months of therapy, as demonstrated by the normalization of the levels of CA125 and PET-CT. We plan to continue olaparib monotherapy for the foreseeable future until disease progression.

Recently, several guidelines, including the US National Comprehensive Cancer Network (NCCN), European Society for Medical Oncology (ESMO), and European Society of Gynaecological Oncology (ESGO), recommend *BRCA1/2* mutations testing upon confirmation of ovarian, fallopian tube, or primary peritoneal cancer, the results of which could be used for maintenance therapy selection following first-line treatment^[11,12]^. With mutation testing earlier in the course of the disease nowadays, hopefully, the treatment for subsequent relapse can be improved. Although our patient did not have germline *BRCA1/2* mutations, she benefitted from the tumor mutation testing, which allowed for precision medicine, an innovative approach to tailoring cancer treatment to specific information of individuals, such as genetic makeup or genetic profile of the tumor. Our patient underwent FoundationOne CDx test, which is an FDA-approved comprehensive genomic profiling to identify patients who may benefit from specific FDA-approved targeted therapies. The test was performed following the failure of multiple lines of chemotherapy for second disease relapse. Retrospectively, if she had the somatic mutations testing earlier in the course of the disease, she could have benefitted from PARPi therapy sooner, without needing to undergo ineffective chemotherapy regimens and their toxicity. Therefore, patients who have disease relapse should be counseled for somatic and germline mutation testing sooner to allow for personalized targeted therapy.

Following treatment with PARPi, a key question is whether our patient should be rechallenged with platinum-based chemotherapy and would she respond to the treatment in the subsequent disease relapse. Germline or somatic mutations in HR genes are found in around one-third of patients with epithelial ovarian cancer^[3]^. One study that investigated somatic and germline mutations in 13 HR genes including *RAD51C* demonstrated that somatic mutations in other HR genes have similar survival outcomes and response rates to platinum-based chemotherapy as germline *BRCA1/2* mutations^[3]^. Theoretically, platinum-based chemotherapy could be considered for our patient in subsequent disease relapse. However, it is unclear whether she could tolerate the toxicities of the treatment in view of poor marrow reserve previously.

In conclusion, our case highlights the potential use of olaparib monotherapy in patients with relapsed ovarian cancer with somatic *RAD51C *mutations when another cytotoxic therapy has failed, as well as the importance of genomic testing to allow for personalized targeted cancer therapy.

## DECLARATIONS

### Copyright

© The Author(s) 2022.
